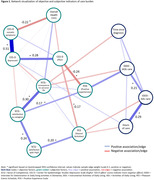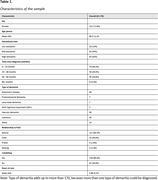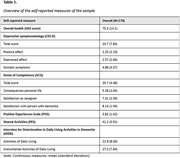# A network approach to dementia care burden: exploring relationships between objective and subjective indicators of care burden in caregivers of community‐dwelling persons with dementia

**DOI:** 10.1002/alz.091248

**Published:** 2025-01-09

**Authors:** Sanne C.E. Balvert, Elke Butterbrod, Sietske A. M. Sikkes, Rose‐Marie Dröes, Erik J. A. Scherder, Maarten V. Milders

**Affiliations:** ^1^ Faculty of Behavioural and Movement Sciences, Vrije Universiteit Amsterdam, Amsterdam Netherlands; ^2^ Alzheimer Center Amsterdam, Neurology, Vrije Universiteit Amsterdam, Amsterdam UMC, Amsterdam Netherlands; ^3^ VU University Medical Center, Amsterdam Netherlands

## Abstract

**Background:**

To support informal caregivers of persons with dementia (PwD), it is fundamental to understand how objective and subjective indicators of care burden are interrelated. This study used psychometric network analyses to explore care burden indicators and extend current models of care in informal caregivers of PwDs.

**Methods:**

Baseline data from an intervention study of 170 informal caregivers of community‐dwelling PwDs was used. Variables included (subscales of) the following measures: a) time since diagnosis, b) objective indicators of care burden: hours of care provided, dependence of the PwD for (Instrumental) Activities of Daily Living (Interview for Deterioration in Daily Living Activities; IDDD), and c) subjective indicators of care burden: caregiver sense of competence (SCQ), positive experiences of caregiving (Positive experiences Scale; POS), shared activities (Pleasant Events Schedule; PES), caregiver depressive symptomatology (Center for Epidemiologic Studies–Depression; CES‐D) and caregiver self‐rated health (VAS‐score). A Gaussian graphical model was employed to explore unique relationships between the measures while accounting for all other relationships in the network. Node strength (strongest total associations of an indicator with other indicators), betweenness and closeness were interpreted if the central stability coefficient was sufficient (>0.25).

**Results:**

Caregivers (71.8% female, 69.5±11.0 years) provided 4.80±5.57 hours of care daily (Table 1 and 2). Node strength and edges reached sufficient stability. The strongest unique relationships existed mostly within objective and subjective measures (e.g., between CES‐D somatic and depressed subscales (r = 0.51); and between total hours of care and ADL‐dependence (r = 0.29), see Figure 1). No significant cross‐connections existed between objective and subjective measures. Within subjective measures, several interrelations were found: PES was associated with SCQ ‐ satisfaction with the PwD (r = 0.20), POS was associated SCQ ‐ satisfaction as caregiver (r = 0.17), and both PES and POS were associated with CES‐D affect (r = ‐0.17, r = ‐0.24). Caregiver’s self‐rated health was only associated with CES‐D somatic (r = ‐0.22).

**Conclusion:**

Our results suggest that objective care burden and disease duration may not be strongly related to caregivers’ experiences, and highlight the interconnectedness of subjective indicators. These insights can contribute to better understanding of care burden and ultimately to more effective support for caregivers of PwD.